# A versatile *cis*-acting element reporter system to study the function, maturation and stability of ribosomal RNA mutants in archaea

**DOI:** 10.1093/nar/gkz1156

**Published:** 2019-12-12

**Authors:** Michael Jüttner, Matthias Weiß, Nina Ostheimer, Corinna Reglin, Michael Kern, Robert Knüppel, Sébastien Ferreira-Cerca

**Affiliations:** Biochemistry III – Institute for Biochemistry, Genetics and Microbiology, University of Regensburg, Universitätsstraße 31, 93053 Regensburg, Germany

## Abstract

General molecular principles of ribosome biogenesis have been well explored in bacteria and eukaryotes. Collectively, these studies have revealed important functional differences and few similarities between these processes. Phylogenetic studies suggest that the information processing machineries from archaea and eukaryotes are evolutionary more closely related than their bacterial counterparts. These observations raise the question of how ribosome synthesis in archaea may proceed *in vivo*. In this study, we describe a versatile plasmid-based *cis*-acting reporter system allowing to analyze *in vivo* the consequences of ribosomal RNA mutations in the model archaeon *Haloferax volcanii*. Applying this system, we provide evidence that the bulge-helix-bulge motif enclosed within the ribosomal RNA processing stems is required for the formation of archaeal-specific circular-pre-rRNA intermediates and mature rRNAs. In addition, we have collected evidences suggesting functional coordination of the early steps of ribosome synthesis in *H. volcanii*. Together our investigation describes a versatile platform allowing to generate and functionally analyze the fate of diverse rRNA variants, thereby paving the way to better understand the *cis-*acting molecular determinants necessary for archaeal ribosome synthesis, maturation, stability and function.

## INTRODUCTION

Ribosomes are universally conserved nanomachines ensuring the decoding of the genetic information encoded within the mRNA into proteins (1). Importantly, ribosome synthesis is one of the most energetically consuming processes in any growing/dividing cell (2,3). Accordingly, ribosome synthesis must be properly regulated and is optimized to appropriately respond to varying environmental cues and organism's lifestyle found across the different domains of life (2,3). Until now, ribosome synthesis has been best studied in bacterial and eukaryotic model organisms (4–9). In contrast, ribosome synthesis in archaea remains to be fundamentally explored (10,11).

Whereas, some ribosome synthesis blueprints are shared across the different domains of life, the apparent complexity of the ribosome biogenesis process has been dramatically expanded in the course of evolution (10,12,13). This increased complexity can be easily highlighted by the plethora of eukaryotic ribosome assembly factors, where >200 factors have been described to be involved in eukaryotic ribosome biogenesis (4,6,8,9,13). In contrast, bacterial ribosome biogenesis requires a condensed subset of ribosome biogenesis factors (7,8,12,14,15). Remarkably and despite the universal conservation of the ribosome, most ribosome biogenesis factors are not conserved between bacteria and eukaryotes (12,13). Accordingly, these observations suggest an intricate evolution history of the ribosome biogenesis process.

Over the last years, several reports support a very intimate relationship between the archaeal and eukaryotic evolutionary history and suggested a deep rooting of the ancestral eukaryotes within the archaeal lineage (16–19). As such, the archaeal phylum potentially appears as a cradle for the early steps of eukaryogenesis. Although this concept is still strongly debated, there are several lines of evidence suggesting that the information processing machineries of archaea and eukaryotes are evolutionary more closely related than their bacterial counterparts (20,21). Therefore, studying how ribosomes are synthesized in archaea may also offer the possibility to shed light on the evolution history of these fundamental processes. Whereas it is difficult to fully predict how ribosome biogenesis may occur in archaea, the presence of a minor fraction of ‘classical eukaryotic’ ribosome biogenesis factors in most archaeal genomes (10,11,13,22) suggests that some aspects of ribosome synthesis may be conserved between archaea and eukaryotes. In agreement with this idea, others and we have shown functional similarities of this process between archaea and eukaryotes *in vitro* and/or *in vivo* (10,11,23–28). Despite these efforts, ribosome biogenesis in archaea still awaits its full molecular characterization.

In the recent years, several studies have described the occurrence of circular-RNA (circ-RNA) in archaea (29–33). Among these circular RNAs, the formation of circ-pre-rRNA intermediates has been suggested in several organisms representative of different archaeal phyla (31,33). These circ-pre-rRNAs were suggested to be generated via the tRNA splicing machinery (31,33,34). The bulge-helix-bulge (bhb) motif present in the 16S and 23S rRNA processing stems which are formed by the hybridization of the sequences flanking the respective mature rRNAs, is presumably used as a cleavage site for the tRNA splicing endonuclease (endA) and subsequently ligated by the tRNA splicing ligase (rtcB), similar to the reactions involved in the maturation of intron-containing tRNAs (10,31,33–35). Remarkably, these observations suggested the existence of a possible unique feature of the archaeal ribosome synthesis pathway. However, the *in vivo* functional relevance of these circ-pre-rRNA intermediates for the formation of mature ribosome has not been investigated so far.

In order to address the functional relevance of these peculiar circular pre-rRNAs for the formation of mature functional ribosomal subunits, and since mutating endA or rtcB may affect both rRNA and tRNA maturation pathways, we sought to mutate rRNA structural elements predicted to be necessary for the formation of these circular pre-rRNAs. To achieve this goal, we have first generated a versatile *cis*-acting element reporter system allowing to analyze the *in vivo* fate of ribosomal RNA mutations, in the model archaeon *Haloferax volcanii* (36). Accordingly, this system was applied to disturb the maturation of the 16S and 23S rRNAs, by generating mutations affecting their respective processing stems; particularly their respective bulge-helix-bulge motifs.

Together, our results provide functional evidence that structural integrity of the respective bulge-helix-bulge motifs and/or the processing stems are required for circ-pre-rRNAs formation and efficient production of stable mature rRNAs in *H. volcanii*. Moreover, we provide evidence for functional coordination during the early phase of rRNA maturation between pre-16S rRNA and pre-23S rRNA processing in *H. volcanii*. In conclusion, our study describes a versatile platform that will allow to generate and functionally analyze the fate of diverse rRNA variants, thereby paving the way to better understand the molecular determinants necessary for archaeal ribosome synthesis, maturation, stability and function.

## MATERIALS AND METHODS

### Strains, plasmids and growth conditions

Strains, plasmids, and oligonucleotides used in this study are listed in Supplementary Tables S1, S2 and S3, respectively.

*Haloferax volcanii* strains (H26 and derivatives) were grown, unless specified, at 42°C under vigorous agitation in *H. volcanii* rich medium (Hv-YPC) or *H. volcanii* enhanced Casamino acids medium (Hv-Ca^+^) (37). *S. acidocaldarius* strain (MW001) was grown in Brock medium supplemented with 180 μM uracil as described previously (38–40).

Molecular cloning and amplification of plasmids were performed according to standard molecular biology methods.

### *H*. *volcanii* transformation

PEG-mediated transformation of H26 strain was performed as described previously (37). Positive transformants were selected on Hv-Ca^+^ lacking uracil.

### Growth analysis of *H. volcanii*

Semi-automated growth analysis was performed as previously described (39). In brief, exponentially growing cells were diluted with fresh medium supplemented with the indicated antibiotic and aliquoted into 96-well plate. Growth (OD_612 nm_) at 41.5°C (±0.3°C) was monitored every 20–30 min for at least 3 days, using a TECAN Infinite F500 reader. Optical density values were corrected with the average background optical density measurement of abiotic medium. Growth analyses for each condition were performed in at least three biological and three technical replicates. Representative results are provided.

### *cis*-acting elements rDNA reporter construction

#### Cloning of the wildtype rDNA locus

The complete rDNA locus (rDNA operon A: HVO_3038-HVO_3042) from *H. volcanii* including ∼700 nucleotides upstream of the 16S rDNA gene and around 40 nucleotides downstream of the tRNA^Cys^ gene was introduced by molecular cloning in three steps into the pTA1228 *H. volcanii* vector (37) generating pRep001 (Supplementary Figure S1). The first three fragments spanning the complete target locus were amplified from genomic DNA by PCR using the following primers: Fragment I: oHv209/oHv155; II: oHv151/oHv154; III: oHv207/208, respectively (see also Supplementary Figure S1). Fragments I–III were respectively cloned into the pCR™-Blunt II-TOPO^®^ (Thermo Fischer Scientific) and sequence integrity verified by DNA sequencing. The obtained fragments were then cloned stepwise into the pTA1228 vector using the corresponding restriction enzymes (Fragment I: KpnI/EcoRV; II: EcoRV/EcoRI; III: EcoRI/NotI) (Supplementary Figure S1). The sequence of the resulting plasmid, pRep001, was verified by DNA sequencing.

#### Generation of cis-acting element rDNA reporter system

Point mutations 16S^A633G^ and 16S^C734T^ (Hv_16S rRNA numbering) and 23S^C2479T^ and 23S^A2496C^ (Hv_23S rRNA numbering) were introduced by site-directed mutagenesis using the following primer pairs oHv221/oHv220, oHv223/oHv222, oHv215/oHv214 and oHv217/oHv216, respectively. Mutations 16S^A633G^ and 16S^C734T^ were first introduced in Fragment I by PCR. The resulting PCR products were cloned into pCR™-Blunt II-TOPO^®^ and sequence integrity was verified by DNA sequencing. Similarly, mutation 23S^C2479T^ and 23S^A2496C^ were introduced in Fragment II and the resulting PCR fragment cloned into cloned pCR™-Blunt II-TOPO^®^. The resulting modified fragments were then sub-cloned into plasmid pRep001, thereby generating the final rDNA *cis*-acting element reporter, pRep002. Sequence integrity was verified by DNA sequencing.

### Mutations of *cis*-acting elements

All mutants (summarized in Supplementary table S2) were generated by site-directed mutagenesis using pRep002 as template.

As described above, the resulting PCR products were first cloned into pCR™-Blunt II-TOPO^®^, verified by DNA sequencing. The corresponding fragments were then sub-cloned into the target vector (pRep002, unless otherwise indicated). Sequence integrity of the reporter region and *cis*-acting mutation(s) was verified by DNA sequencing.

### *cis*-acting element reporter assay

Standard analysis workflow is summarized in Supplementary Figure S2. All analyses were performed at least in biological duplicates (two independent transformants) and technical quadruplets (two fluorescent channels and two independent quantitation).

#### In culture PCR

H26 transformed with the indicated plasmids were grown in Hv-Ca^+^ lacking uracil to OD_600nm_ = 0.6–0.8. To screen positive clones and to quantify the template ratios of plasmid/genomic DNA for later normalization, 50 μl of the culture were diluted in 950 μl H_2_O. One microliter of the dilution was used as template for PCR with the fluorescently labeled primers oHv305/oHv306 (16S rDNA) and oHv322/oHv323 (23S rDNA). Resulting PCR products were purified via Na-Acetate/EtOH precipitation, and subsequently digested with the respective restriction enzymes EcoRV-HF (16S rDNA) or BssSI (23S rDNA) (see below).

#### Preparation of total RNA

H26 transformed with the indicated plasmids were grown in Hv-Ca^+^ lacking uracil to OD_600nm_ = 0.6–0.8. Two ml of cells were aliquoted and pelleted by centrifugation (6000 rpm; 8 min) and stored at −20°C for later usage or total RNA was immediately extracted. Total RNA was extracted using the hot-phenol extraction procedure as previously described (39,41). The extracted RNA was resuspended in 50 μl RNase free H_2_O and DNase treated with DNAse RQ1 (Promega) in presence of RNAse inhibitor (Promega) at 37°C overnight. DNase-treated RNA was precipitated with Na-Acetate/EtOH. RNA concentrations were estimated on the Nanodrop.

#### cDNA synthesis

Synthesis of cDNA was performed using Superscript™ III reverse transcriptase (Thermo Fisher Scientific) according to the manufacturer′s protocol. In brief, 1–2 μg DNase-treated RNA was used for the reverse transcriptase reaction. Note that no reverse transcriptase controls were performed for every single analysis to ensure that no DNA contamination is remaining for the downstream analysis. To initiate reverse transcription the following oligonucleotides have been used: for circular-pre-rRNA intermediates detection oHv40 (16S rRNA) and oHv42 (23S rRNA) respectively, and oHv252 (16S rRNA) and oHv307 (23S rRNA) for total rRNA amounts determination.

#### Polymerase chain reaction

The amplification of 16S and 23S ribosomal RNA target sequences was performed using 1 μl of the synthesized cDNA as template. PCRs were performed as described above. PCRs including no RT control, were analyzed on agarose gels. Samples showing remaining amounts of DNA contaminations or inefficient PCR amplification were discarded and repeated. PCR products were precipitated by EtOH/Na-Acetate precipitation and resuspended in 25 μl H_2_O.

#### Restriction digest

After precipitation, similar amounts (∼150 ng) of PCR products were digested in a total volume of 30 μl, with 20 U and 10 U of the respective enzymes. PCR products within the 16S rDNA locus were digested with EcoRV-HF (NEB) for 4 h, PCR products within the 23S rDNA locus were digested with BssSIα (NEB) overnight. To ensure complete digestion, PCR products using pRep002 as template and encompassing the respective restriction digest sites were used as control (Supplementary Figure S2). The digested samples were then separated using a TBE–10% Polyacrylamide gel. Fluorescent signals were detected using a Li-COR Odyssey Imaging system.

#### Detection and quantification

Quantification of all obtained images was done with Fiji (42). Signals derived from the fluorescently labeled PCR primers were detected at 700 and 800 nm. The intensities of the gel bands were determined in two independent measurements for both wavelengths, respectively. After correction of every value with the corresponding background value, the ratios between plasmid and genomic derived rRNA or rDNA amounts were calculated. The rRNA ratios were normalized on their respective DNA template amounts.

### Circular pre-rRNA intermediates identification

RNA from logarithmically growing *H. volcanii* (H26) and *S. acidocaldarius* (MW001) cells were extracted and DNase-treated as described previously (39). Reverse transcriptase reactions were performed using primers oHv040/oHv042 and Saci009/Saci014 using Superscript™ III according to manufacturer′s recommendations. Circular pre-rRNA region was amplified using divergent PCR using primers oHv040/oHv039 (Hv_circ-pre-16S rRNA), oHv041/oHv042 (Hv_circ-pre-23S rRNA), and Saci009/Saci010 (Saci_circ-pre-16S rRNA) Saci015/Saci014 (Saci_circ-pre-23S rRNA). Ligation extremities were determined based on permutated sequence obtained by DNA sequencing. 5′extended pre-rRNA intermediates were amplified with oHv040/oHv200 (Hv_5′extended-pre-16S rRNA) oHv042/oHv201 (Hv_5′extended-pre-23S rRNA) and Saci009/Saci013 (Saci_5′extended-pre-16S rRNA) Saci014/Saci016 (Saci_5′extended-pre-23S rRNA).

Resistance of circular pre-rRNA to exonuclease activity of RNAse R was performed as following. DNase-treated total RNA was incubated with or without RNase R (Epicenter) at 37°C for 2 h. RNase R-treated total RNA was purified by hot-phenol extraction and subjected to cDNA synthesis as described above. Quantitative RT-PCR analysis was performed with Sybr-green, using primer pairs amplifying linear pre-16S/23S and circular-pre16/23S rRNA as described above. Relative quantification analysis was performed using the comparative analysis software module provided by the manufacturer (Rotor-gene 6 – Corbett Research/Qiagen). Relative amounts of linear/circular pre-rRNA were determined according to the 2^−ΔΔC^_T_ method (43). For comparison, the ratio of linear/circular pre-rRNA obtained in the non-RNase R-treated sample was arbitrarily set to one. Experiments were performed in biological replicates and serial dilutions of the samples were run in triplicates to ensure the accuracy of the data.

### Ribosomal RNA operon organization

Ribosomal RNA operon organization was deduced from the information provided at the UCSC archaeal genome browser (http://archaea.ucsc.edu/) (44), and previous studies (11,45–47).

### RNA structure prediction

Sequences flanking the mature 16S and 23S rRNA were retrieved from UCSC archaeal genome browser (http://archaea.ucsc.edu/) (44). Iterative structure prediction using different length of sequence input was performed with the ViennaRNA web servers (48).

## RESULTS

### Characterization of circular-pre-rRNAs in *Haloferax volcanii* and *Sulfolobus acidocaldarius*

A pioneering RNomics study of two evolutionary divergent archaea (*Archaeoglobus fulgidus* and *Sulfolobus solfataricus)* has initially suggested the presence of circular pre-rRNA intermediates (33). More recently, a systematic and targeted analysis of RNA circularization in several organisms representative of two major archaeal groups (*Sulfolobus solfataricus*, *Sulfolobus acidocaldarius* and *Halobacteriumsalinarum*, respectively) has experimentally confirmed the presence of these circ-pre-rRNA intermediates in archaea (31). These circularization events can be detected by (i) targeted-divergent PCR analysis and (ii) resistance of the corresponding circular RNA candidate to treatment with Ribonuclease R (RNase R) (31).

**Figure 1.**
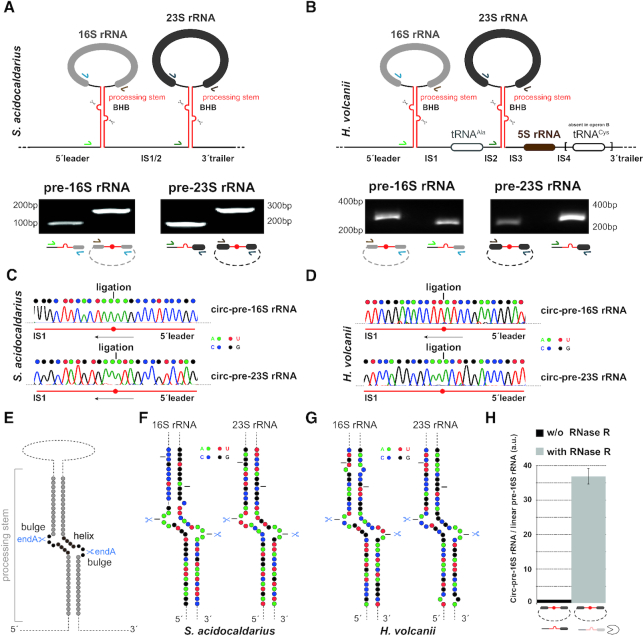
Characterization of circular pre-rRNA *in H. volcanii* and *S. acidocaldarius*. (**A**) Detection of circular pre-rRNA intermediates in *S. acidocaldarius*. The unique rDNA operon present in *S. acidocaldarius* (Saci_1299-1300 and flanking regions) is schematically represented (44,110). The double-stranded processing stems containing the bulge-helix-bulge motif, putative substrate of the archaeal tRNA processing machinery are indicated (upper panel). 5′extended linear 16S/23S pre-rRNA and circular pre-16S/23S pre-rRNA intermediates were amplified by RT-PCR and analyzed by agarose gel electrophoresis (lower panel). Reverse transcriptase was performed using reverse complement primers hybridizing at the 5′end of the mature 16S and 23S rRNA (depicted in light and dark blue, respectively). The 5′extended linear 16S/23S pre-rRNA were amplified using the indicated primer pairs (depicted in light and dark blue, light and dark green) respectively. Circular pre-16S/23S pre-rRNA intermediates were amplified using the indicated primer pairs (indicated in light and dark brown, and in light and dark blue, respectively). Expected sizes of the PCR products are as following: 5′extended linear 16S rRNA (primers Saci009/013) 98 bp; 5′extended linear 23S rRNA (primers Saci014/016) 168 bp; circular pre-16S rRNA (primers Saci009/010) 176 bp and Circular pre-23S (primers Saci014/015) 271 bp. (**B**) Detection of circular pre-rRNA intermediates in *H. volcanii*. Same as in (A), one (operon A: HVO_3038-HVO_3042 and flanking regions) of the two rDNA operons present in *H. volcanii* and characterized by the presence of an additional tRNA^Cys^ at its 3′end is schematically depicted (44,64). Reverse transcriptase was performed using reverse complement primers hybridizing at the 5′end of the mature 16S and 23S rRNA (indicated in light and dark blue, respectively). The 5′extended linear 16S/23S pre-rRNA were amplified using the indicated primer pairs (depicted in light and dark blue, light and dark green) respectively. Circular pre-16S/23S pre-rRNA intermediates were amplified using the indicated primer pairs (depicted in light and dark brown, and in light and dark blue, respectively). Expected sizes of the PCR products are as following: 5′extended linear 16S rRNA (primers oHv200/40) 235 bp; 5′extended linear 23S rRNA (primers oHv201/42) 279 bp; Circular pre-16S rRNA (primers oHv39/40) 333 bp and Circular pre-23S 208 bp. (**C**, **D**) Determination of ligation extremities by DNA sequencing. Sanger sequencing chromatogram and the corresponding deduced nucleotide sequences of the region surrounding the RNA ligation position as determined by DNA sequencing of the PCR product obtained in (A, B) are provided for *S. acidocaldarius* (C) and *H. volcanii* (D), respectively. Nucleotides are color-coded as indicated in the figure (A: green; U: red; C: blue; G: black). (**E**) Schematic representation of rRNA processing stem. The bulge-helix-bulge motif and expected splicing endonuclease (endA) cleavage sites are indicated. (**F**, **G**) Schematic representation of *S. acidocaldarius* (F) and *H. volcanii* (G) rRNA processing stems. 2D structure prediction of the respective processing stems was determined using the ViennaRNA web services (RNAcofold and RNAfold servers - http://rna.tbi.univie.ac.at/) (48) using default parameters. Nucleotides are color-coded as indicated above and in the figure. EndA cleavage sites within the respective bulge-helix-bulge motifs are indicated by scissors. The black lanes delineate the sequence boundaries shown in panel (C, D). (**H**) Relative abundance of circular-pre-rRNA after RNase R treatment. DNase-treated total RNA obtained from *H. volcanii* was treated with RNAse R to eliminate linear RNA prior to cDNA synthesis and quantitative PCR analysis (see Materials and Methods). The relative abundance of linear pre-rRNA versus circular pre-rRNA as determined by qPCR analysis was normalized to the non-RNAse R treated samples and arbitrarily set to one. The depicted results were obtained from analysis performed in biological duplicates and technical triplicates.

Based on these observations, we aimed to show the presence of circular-pre-rRNAs in the genetically tractable model archaeal organism, the Euryarchaeota *H. volcanii*. As a positive control of our analysis, we have used the Crenarchaeota *S. acidocaldarius*, for which circular-pre-rRNAs have been demonstrated previously (31). We have first performed circular-pre-rRNA-specific divergent RT-PCR analysis. As shown in Figure 1, we could confirm the presence of both circ-pre-16S rRNA and circ-pre-23S rRNA intermediates in these two model archaea (Figure 1A and B). In order to determine the position of the ligated extremities of these pre-rRNA intermediates, we have cloned the respective rRNA-specific divergent PCR products and determined their respective sequences (Figure 1C and D). Accordingly, we could map the cleavage and ligation events within the pre-rRNA spacer sequences flanking the mature rRNA sequences (Figure 1E and G). In agreement with previous studies (31,33), all cleavage and ligation events faithfully mapped to the bulge-helix-bulge motifs present in the processing stems formed by the hybridization of the pre-rRNA spacers surrounding the mature rRNAs (Figure 1E and G). Finally, and as expected for circular RNA, these pre-rRNA intermediates showed increased stability towards RNAse R treatment as opposed to linear pre-rRNA intermediates (e.g. in Figure 1H) (31).

Together, our results further confirm and extend the widespread prevalence of circ-pre-rRNA intermediates in representative archaeal model organisms.

### Towards an rDNA *cis*-acting element reporter assay

Next, we aimed to analyze the functional relevance of these circ-pre-rRNA intermediates for the synthesis of mature rRNAs. However, functional perturbation of the only known putative *trans*-acting factors (endA & rtcB) involved in this circularization step, may potentially lead to a pleiotropic effect influencing both intron-containing tRNA and pre-rRNA maturation pathways. Moreover, due to their central role in rRNA and tRNA maturation and in agreement with recent studies, these genes were expected to be essential for cell viability (49,50). Therefore, we decided to directly mutate key *cis*-acting elements, like the respective bulge-helix-bulge motifs and/or the processing stems and analyze the consequences of these mutations on the formation of circ-pre-rRNAs and mature functional ribosomal subunits. To achieve such analysis, we have designed a plasmid-based system allowing, on the one hand the easy and fast generation of viable/unviable rRNA mutants, and on the other hand, to follow the fate of the mutated (pre-)rRNAs in a qualitative and quantitative manner. Whereas such plasmid-based *cis*-acting element rDNA reporter systems have been exploited to various extent in bacteria and eukaryotes (51–63), up until now no comparable system has been fully harnessed in archaea.

Mutations of important *cis*-acting elements are expected to affect (pre-)rRNA production in a quantitative and/or qualitative manner (e.g. either reduced production of rRNA, or production of non-functional, and/or non-fully processed, and/or unstable rRNA intermediates). Therefore, to elucidate the contribution of diverse (pre-)rRNA *cis*-acting elements to ribosome formation and function we aimed to generate a well applicable reporter assay allowing to distinguish between the different possibilities described above.

**Figure 2.**
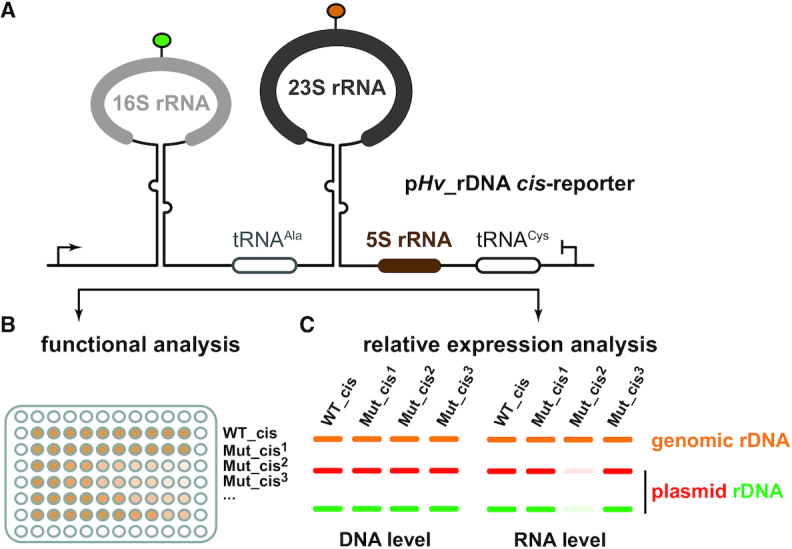
General features of the rDNA *cis*-acting element reporter system. (**A**) Schematic representation of the plasmid-based rDNA *cis*-acting element reporter system used in *H. volcanii*. Modified ribosomal DNA operon A from *H. volcanii* cloned into pTA1228 vector is depicted. The rDNA modifications aim to include a read-out system allowing to score the relative functionality by means of partial antibiotic resistance (**B**) and the relative expression level of plasmid-born/genomic (pre-)rRNA intermediates (**C**). The latter is achieved by (RT-)PCR analysis over a common region for which one or two nucleotide exchanges provide a mean to disentangle the allelic origin (genomic vs plasmid-born) of the amplified fragment by restriction enzyme analysis (see Materials and Methods and text for details).

Based on previous *cis*-acting element reporter systems generated either in bacteria and/or in eukaryotes (52,56,58,59,61,62) and in order to obtain a functional status of the plasmid-encoded rRNA variants, we sought to take advantage of antibiotic resistance as read-out assay for ribosomal subunit variant functionality. In addition, we sought to use an ‘RNA-tagging’ strategy to introduce differences that would enable to quantitatively follow and discriminate between the plasmid-encoded (pre-)rRNA variants from the two chromosomally expressed wildtype rRNA gene loci present in *H. volcanii* (64). A general outline of the experimental approach reflecting the requirements described above is depicted in Figure 2. In order to fulfill the criteria above, we sought to develop a plasmid-born engineered rDNA locus (Figure 2A) that could confer (i) rDNA expression-dependent antibiotic resistance (Figure 2A) and (ii) a semi-quantitative and allele-specific readout (Figure 2C). The latest is achieved by (RT-)PCR analysis over a common region for which one or two nucleotide exchanges provide a mean to disentangle the allelic origin (genomic versus plasmid-born) of the amplified fragment by restriction enzyme analysis (see below for details).

### Screening for antibiotics affecting growth of *Haloferax volcanii*

**Figure 3.**
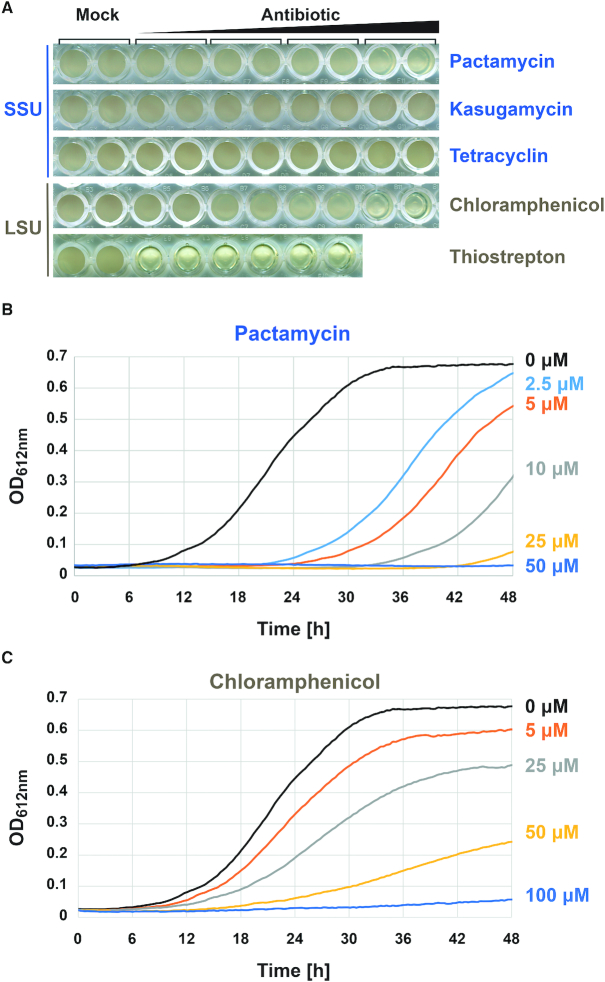
Selection of antibiotics inhibiting *H. volcanii* growth. (**A**) Growth analysis of *H. volcanii* in presence of various antibiotics. Growth of *H. volcanii* in Hv-YPC and in presence of increasing amounts of various antibiotics targeting either the small ribosomal subunit (SSU – indicated in blue) or large ribosomal subunit (LSU – indicated in gold) was monitored using a semi-automated plate reader system (see Material and Methods). End point picture (2 days) of an exemplary analysis is provided. Note the decreased turbidity in presence of Thiostrepton or the highest concentration of Pactamycin and Chloramphenicol used in these conditions. (**B**, **C**) Exemplary growth curve of *H. volcanii* cells incubated with the indicated amounts of Pactamycin (B) or Chloramphenicol (C). Concentration-dependent growth inhibition using the indicated antibiotics was monitored in a 96-well plate format over 48 h. Representative results are provided.

Accordingly, we first aim to identify suitable antibiotics affecting both ribosomal subunits. Archaea are generally believed to be poorly affected by antibiotics commonly used against bacteria (65). However, previous studies suggested that a subset of molecules are partly or fully inhibiting archaeal growth in a cell type-dependent manner (66–70). After reviewing the literature for potential antibiotics affecting growth of haloarchaea and considering the known target and molecular mechanisms of resistance, we selected several antibiotics affecting either the small ribosomal subunit (SSU) or the large ribosomal subunit (LSU). Concentration-dependent inhibition of cellular growth was systematically analyzed using a previously described semi-automated growth analysis (25,39) and is summarized in Figure 3. Among the various antibiotics showing concentration-dependent inhibition of *H. volcanii* growth, we decided to focus on the use of Pactamycin (Figure 3B) and Chloramphenicol (Figure 3C) which target the SSU and LSU, respectively (68,69) and presented a broad concentration range of growth inhibition (Figure 3).

### Plasmid-based expression of engineered rRNA in *H. volcanii* confers partial resistance to Pactamycin and Chloramphenicol

**Figure 4.**
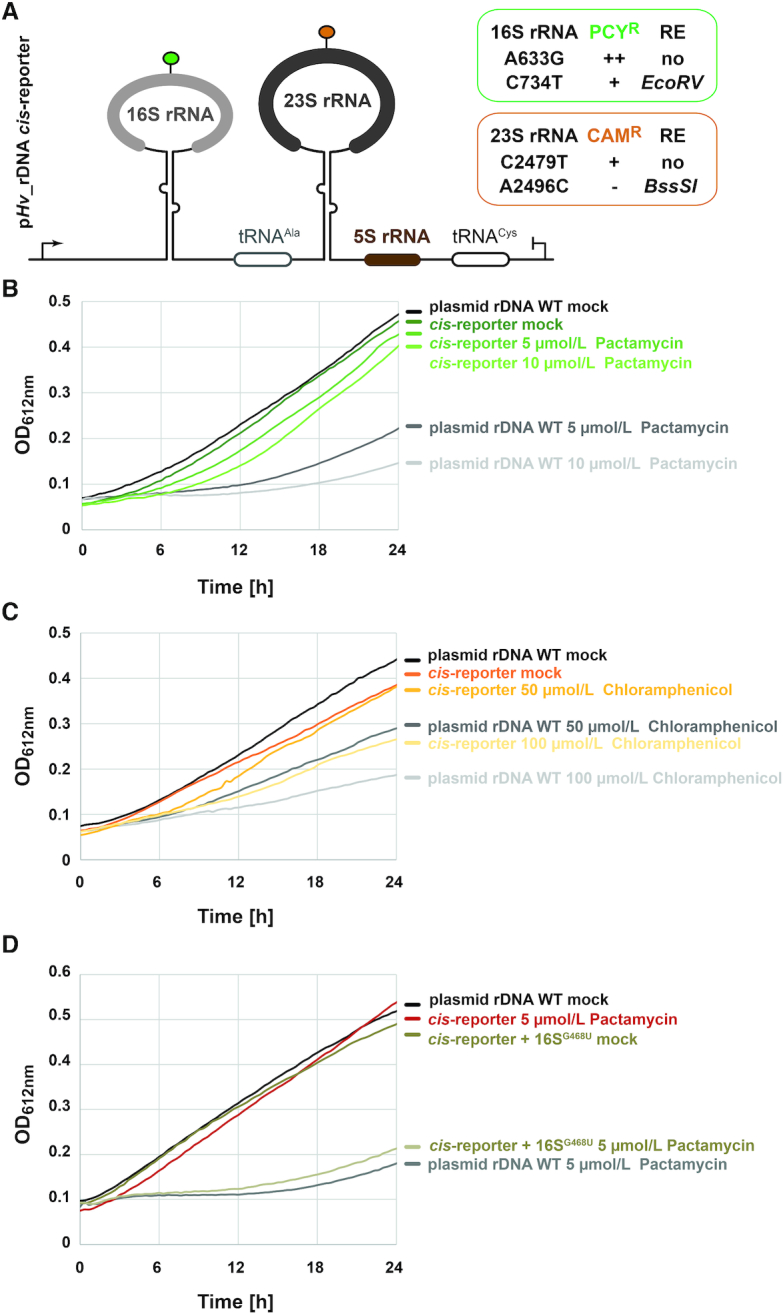
Plasmid-based engineered rDNA provides partial antibiotic resistance to *H. volcanii* cells. (**A**) Schematic representation of the plasmid-based rDNA *cis*-acting element reporter system used in *H. volcanii*. Indicated modifications expected to confer Pactamycin resistance (PCY^R^) and Chloramphenicol resistance (CAM^R^) were introduced in the rDNA sequence by molecular cloning (see text and Materials and Methods for details). Note that the individual changes provided different degrees of antibiotic resistance and are summarized in the respective boxes (data not shown). Changes in sequence generating new restriction enzyme digestion sites (RE) are also indicated. (**B**) Ribosomal RNA *cis*-acting element reporter provides partial resistance to Pactamycin. Cells transformed with a plasmid bearing unmodified rDNA operon A (level of gray) or the rDNA *cis*-acting reporter (level of green) were grown in Hv-Ca^+^ lacking uracil and supplemented with the indicated amounts of Pactamycin. Growth was monitored as described above. Note that the presence of the *cis*-acting element reporter provides growth advantage in presence of Pactamycin in comparison to wildtype control. Representative results are provided. (**C**) Ribosomal RNA *cis*-acting element reporter provides partial resistance to Chloramphenicol. Same as is (B), except that cells were grown in presence of Chloramphenicol (level of orange). Note that the presence of the *cis*-acting element reporter provides growth advantage in presence of Chloramphenicol in comparison to wildtype control. Representative results are provided. (**D**) Partial antibiotic resistance depends on rRNA functional integrity. The *cis*-acting element reporter system was further modified by addition of a non-functional mutation affecting the SSU decoding center (16S^G468U^ – *H. volcanii* numbering). Cells were transformed with the wildtype *cis*-acting element reporter and the 16S^G468U^ mutated *cis*-reporter. Growth in presence of Pactamycin was monitored as described above. Note that the non-functional 16S^G468U^ mutation abolishes the antibiotic resistance initially provided by the *cis*-acting element reporter plasmid.

Encouraged by the concentration-dependent inhibition of cellular growth of *H. volcanii* described above, we cloned one of the two rDNA operons (HVO_3038-HVO_3042) and its flanking regions into an *E. coli*–*H. volcanii* shuttling vector (see Supplementary Figure 1, Materials and Methods for details). In addition, we introduced various mutations expected to sustain ribosome functionality while providing resistance against Pactamycin and Chloramphenicol, respectively (66,68,69). The derived plasmids carrying the respective rDNA variants were transformed into host wildtype *H. volcanii* cells (H26) and selected on *H. volcanii* enhanced casamino acids medium lacking uracil (Hv-Ca^+^) (37). Antibiotic resistance of the respective transformants was analyzed in presence of increasing amounts of antibiotics as described above and is summarized in Figure 4A. Mutations in the 16S rRNA at position A633G (16S^A633G^; *H. volcanii* numbering, unless otherwise stated) and at position C734T (16S^C734T^) conferred various degrees of Pactamycin resistance to cells expressing these rRNA variants (Figure 4A). Similarly, cells expressing rRNA variant 23S rRNA at position C2479T (23S^C2479T^) were partly resistant to Chloramphenicol, whereas mutation in the 23S rRNA A2496C (23S^A2496C^) did not provide any measurable growth advantage in the presence of Chloramphenicol in the experimental conditions tested (Figure 4A). The four mutations mentioned above where intragenetically combined (pRep002, hereafter described as *cis*-reporter). The mutations combination provided similar resistance properties against Pactamycin (Figure 4B) and Chloramphenicol (Figure 4C) as observed for the individual mutations providing the strongest antibiotics resistance (16S^A633G^ and 23S^C2479T^, respectively) (data not shown). Whereas, the additional mutations (16S^C734T^ and 23S^A2496C^) provided substantial advantages for additional quantitative analysis (see below for full description).

To further support the possibility to score for rRNA variants functionality based on relative antibiotic resistance, we introduced an additional mutation within helix 18 (Hv 16S^G468U^/Ec 16S^G530U^) which participates in the functional establishment of the SSU decoding center (58,71,72). As expected for a mutation affecting ribosome function, partial resistance to Pactamycin provided by the *cis*-reporter was lost at the concentrations of Pactamycin analyzed (Figure 4D).

Together, our results suggest that plasmid-based engineered rDNA is functionally expressed in *H. volcanii* and that its relative functionality status can be measured by means of partial antibiotic resistance conferred by the presence of additional point mutations in our engineered *cis*-reporter system.

### Semi-quantitative analyses of (pre-)rRNA variants expression

**Figure 5.**
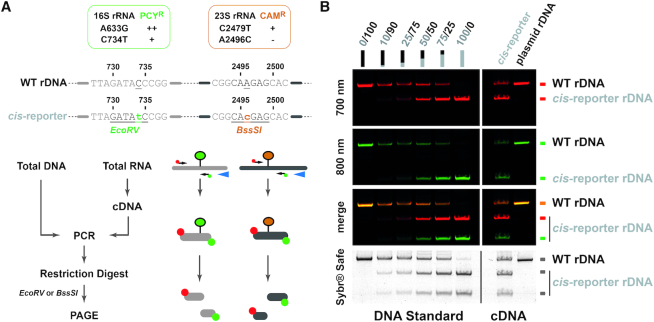
Relative expression analysis of endogenous rRNA and plasmid-derived rRNA using differential RT-PCR/restriction digest. (**A**) Principle of differential (RT-)PCR/restriction digest analysis. General properties (antibiotic resistance/additional restriction enzyme digestion sites) conferred by the plasmid-based rDNA *cis*-acting element reporter system used in *H. volcanii* is summarized (upper panel). General strategy to analyze relative amounts of rDNA/rRNA levels is schematically represented (lower panel). The reporter assay takes advantage of additional restriction digest sites to distinguish between the relative amounts of endogenous and plasmid-derived rDNA/rRNA using fluorescently labeled PCR primers and restriction digest. Note that undigested PCR products stem from the endogenous rDNA/rRNA and the digested products from the plasmid-based rDNA *cis*-reporter. The ratio of digested/undigested product provides a semi-quantitative estimation of plasmid-based/endogenous rDNA/rRNA levels. (**B**) The rDNA *cis*-acting reporter system is expressed *in vivo* and its expression can be distinguished from the endogenous population. A DNA standard containing varying ratio of plasmids carrying the wildtype rDNA or the rDNA *cis*-acting reporter was used as PCR template and restriction digest analysis to evaluate the relative expression of the rDNA *cis*-acting element reporter (left panel – DNA Standard). DNase-treated total RNA extracted from cells transformed with a plasmid carrying either wildtype rDNA (plasmid rDNA) or the *cis*-acting element reporter (*cis*-reporter) was subjected to RT-PCR/restriction digest analysis (right panel – cDNA). Digested PCR products were separated by PAGE. Fluorescence signals (700 and 800 nm) were acquired using a Li-COR Odyssey system. Bulk DNA was then visualized by Sybr-safe staining.

During the molecular cloning steps of our *cis*-acting element reporter system, we noticed that mutations 16S^C734T^ and 23S^A2496C^ created additional EcoRV and BssSI restriction sites, respectively (Figure 5). Accordingly, we decided to evaluate the possibility to take advantage of these minor sequence changes to follow the relative amount of rDNA variants and the relative expression of plasmid-encoded (pre-)rRNA variant compared to the genomically expressed (pre-)rRNA. To this end, we set-up a read-out system based on reverse transcriptase/PCR amplification reaction and restriction enzyme digestion allowing to distinguish between genomically expressed wildtype rRNA and plasmid-expressed rRNA variant(s) (Figure 5 and below). Moreover, to facilitate visualization and quantification, we have used differentially fluorescently labelled oligonucleotides during the PCR reaction. In such set-up direct PCR/restriction enzyme-based analysis performed with cellular DNA virtually delivers information on the relative amounts of genomically-encoded/plasmid-encoded rDNA. In addition, PCR/restriction enzyme-based analysis from cDNA provides information on the relative amounts of genomically-expressed/plasmid-expressed rRNAs (Figure 5 and below). Together, such analysis allows estimation of the relative expression levels of the respective rRNA variants normalized to the amounts of DNA template available in the cells. As shown in the exemplary analysis (Figure 5), relative expression of genomically- and plasmid-encoded/-expressed rDNA/rRNA variants can be determined.

Overall, the *cis*-acting element reporter system described above allows relative quantitative and qualitative analysis of rRNA variants in the model archaeon, *H. volcanii* (see below).

### *cis*-acting element reporter system is not subjected to massive recombination events

**Figure 6.**
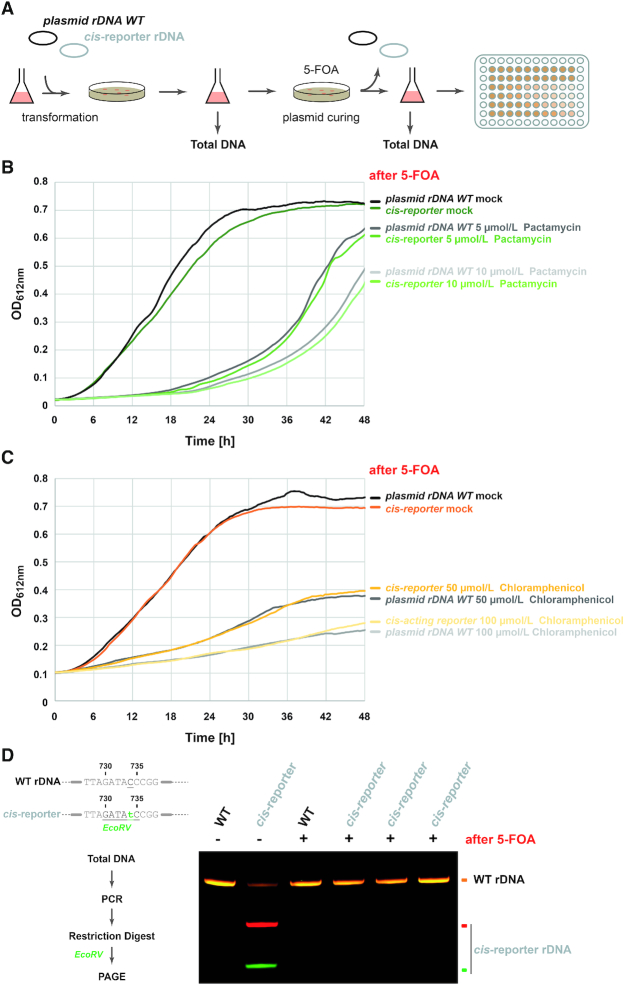
Plasmid rDNA *cis*-acting element reporter is stably propagated in wildtype H26 cells. (**A**) Experimental strategy to assess genomic recombination events of the rDNA *cis*-acting element reporter. Properties of cells (antibiotic resistance/ restriction digest site) transformed with the rDNA *cis*-acting reporter plasmid were analyzed before and after curing the *cis*-reporter plasmid with 5-FOA. (**B**, **C**) Growth analysis of cells cured from the *cis*-acting reporter system. Cells transformed with either a plasmid containing the wildtype rDNA, or the *cis*-acting reporter were plated on 5-FOA containing plates to promote plasmid loss. Independent clones were selected and grown in presence of Pactamycin (B) or Chloramphenicol (C). Representative analysis is depicted. Similar experimental outcome was observed for all individual clones tested (data not shown). (**D**) Analysis of persistence of additional restriction digest site after plasmid-loss. Cells transformed with either a plasmid containing the wildtype rDNA, or the *cis*-acting reporter were plated on 5-FOA containing plates to promote plasmid loss. Independent clones were selected for PCR/restriction digest analysis using the 16S rDNA amplicon and analyzed by PAGE as described above. No significant recombination of the *cis*-acting reporter plasmid with genomic DNA could be detected as indicated by the absence of digested PCR product in the 5-FOA^R^ clones. Representative analysis is provided. Similar experimental outcome was observed for all individual clones tested (data not shown).

A possible disadvantage of such a reporter strategy can arise from recombination events which could potentially exchange DNA information between plasmids/genomically encoded rDNA, thereby introducing a possible bias for accurate functional and quantitative analysis. To exclude this possibility, we cured the cells transformed with our *cis*-acting element reporter using 5-FOA (73) which selects for cells losing the plasmid-encoded selection marker *pyrE2* (Orotate phosphoribosyltransferase encoded in the *cis*-reporter plasmid) and analyzed the loss of growth in the presence of antibiotic and by the absence of plasmid-specific additional restriction site in the 5-FOA treated cells (Figure 6A). As shown in Figure 6, cells treated with 5-FOA and now lacking the *cis*-acting element reporter were not anymore resistant to the respective antibiotics and showed similar antibiotic inhibition when compared to the parental strain (Figure 6B-C). Moreover, analysis on the rDNA population demonstrated that plasmid loss and recovery of antibiotic sensitivity correlates with the absence of additional *cis*-acting reporter specific restriction sites (Figure 6D and data not shown). Together, these results provide further evidence that our developed *cis*-acting element reporter is not massively recombining with the genome of the host cells and thus allows a faithful, relative quantitative and qualitative analysis of (pre-)rRNA variants.

### Mutations of bulge-helix-bulge motif/processing stem affect formation of circ-pre-rRNA and mature rRNA

**Figure 7.**
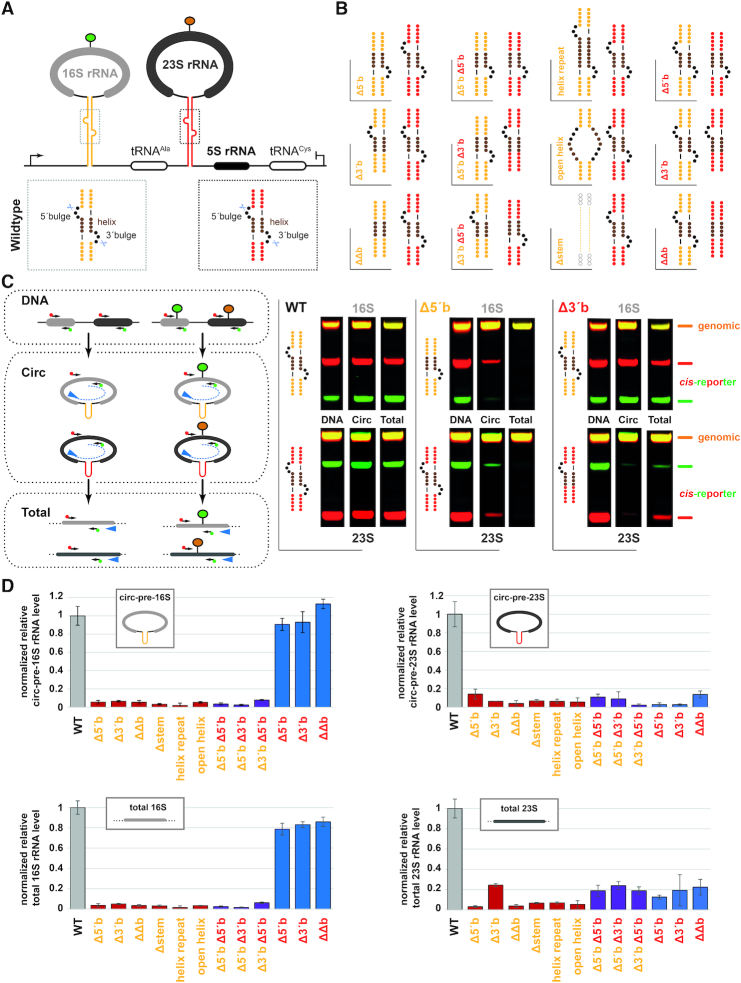
Role of the bulge-helix-bulge motif for the synthesis of circular-pre-rRNA and total rRNA. (**A**) Schematic representation of the rDNA *cis*-acting reporter system. The 16S and 23S rRNA processing stems containing the bulge-helix-bulge motif are depicted in yellow and red, respectively. A close-up representation (dashed-line box) indicates the structural elements that were subjected to site-directed mutagenesis (see below). The tRNA splicing endonuclease cleavage sites are indicated by scissors. (**B**) Structure predictions of processing stem mutants analyzed in this work. The indicated processing stem mutants collection was generated by molecular cloning and transformed into wildtype *H. volcanii* cells as described in Material and Methods. The predicted structural consequences (ViennaRNA webservers) of the respective mutations introduced in the rDNA *cis-*acting reporter are depicted. (**C**) Bulge-helix-bulge motif/processing stem integrity is required for efficient circular-pre-rRNA and total rRNA formation. Cells transformed with the *cis*-reporter carrying the additional mutations depicted in (B) were analyzed using the schematically depicted work flow (left panel). In brief, relative total DNA amounts (plasmid-based/endogenous rDNA) were analyzed by PCR/restriction digest analysis (upper left panel). Relative amounts of circular-pre-rRNA were determined by RT-PCR/restriction digest analysis using a reverse transcription primer (blue arrow) positioned at the 5′end of the respective 16S or 23S rRNAs and extending through the rRNA region subjected to PCR amplification only when circularized (middle left panel). Relative total rRNA amounts (essentially corresponding to mature rRNA) were obtained by RT-PCR/restriction digest analysis using the indicated primers (blue arrow indicates the reverse transcription primer used). PCR fragments were digested (16S: *EcoRV*; 23S: *BssSI*) and separated by PAGE. Fluorescent signals were acquired on a Li-COR Odyssey system. Exemplary PAGE analysis of wildtype, 16S 5′bulge deletion and 23S 3′bulge deletion is provided. Note that the relative rDNA amounts are similar, independent of the mutations examined. (**D**) Early steps of rRNA maturation are functionally coordinated in *H. volcanii*. Results of semi-quantitative relative expression analysis of 16S/23S circular-pre-rRNAs (upper panel) and total 16S/23S rRNAs (lower panel) obtained from the processing stem mutants analysis depicted in (B) are summarized. Relative ribosomal RNA expression was normalized to the relative amounts of the respective rDNA template (plasmid-based versus endogenous) and is expressed in comparison to the wildtype situation (arbitrarily set to one) (see Materials and Methods for details). Each rDNA variant analysis was performed at least in biological duplicates (two independent transformants) and technical quadruplets (two fluorescent channels and two independent quantifications)

With our *cis*-acting element reporter at hands, we went back to our initial biological question aiming to characterize the functional relevance of circ-pre-rRNA intermediates for the synthesis of mature rRNAs. Hence, we generated a collection of various mutations modifying the bulge-helix-bulge motifs and/or the processing stems integrity of the 16S and 23S rRNA, respectively (Figure 7A and B). These mutants were analyzed according to the general strategy outlined in Supplementary Figure S2. In brief, at least two independent transformants were selected and characterized at the DNA and RNA level using the rDNA/(pre-)rRNA specific fluorescently labelled (RT-)PCR approach (see above). Moreover, to provide information about the relative amounts of circular pre-rRNA generated in these conditions, we modified our cDNA synthesis/PCR, restriction enzyme analysis to allow specific determination of the relative steady-state level of these circ-pre-rRNA intermediates (Figure 7C).

All the different *cis*-acting element plasmids tested (depicted in Figure 7B) were present at similar relative DNA levels (Figure 7C and data not shown). As summarized in Figure 7, mutations affecting either the bulge-helix-bulge motifs or the processing stems integrity showed decreased relative amounts of their respective circ-pre-rRNA and total rRNA population (Figure 7C and D). Remarkably, whereas mutations affecting the 23S rRNA processing stem showed a specific decrease of the 23S total rRNA population including circ-pre-23S rRNA (Figure 7C and D), mutations of the 16S processing stem showed decrease of both the 16S and 23S total rRNA population, including 16S and 23S rRNA circ-pre-intermediates.

In summary, these results suggest that the processing stem and bulge-helix-bulge motif integrity is required for efficient circular pre-rRNA intermediates and total rRNA formation. Moreover, our analysis also suggests a mechanism by which early steps of 16S pre-rRNA maturation influence the efficient formation of circ-pre-23S rRNA and total 23S rRNA. Remarkably, the opposite effect was not observed in the conditions tested. Finally, our investigation suggests that early steps of pre-rRNA processing are functionally coordinated in *H. volcanii*. However, the exact underlying molecular mechanisms of this intriguing coordination remain to be determined (see Discussion).

## DISCUSSION

### A general reporter assay for the analysis of (pre-)rRNA *cis*-acting element in archaea

In this work, we describe the development and application of a versatile *cis*-acting reporter assay allowing to functionally characterize (pre-)rRNA *cis*-acting element perturbation in the model archaeon, *H. volcanii*. This system provides a relatively straight forward qualitative and quantitative read-out system enabling to determine the functional consequences of (pre-)rRNA mutations on the maturation, stability and function of ribosomal subunits *in vivo*.

The molecular ‘life and death’ of ribosomal subunits in archaea is still poorly characterized (10,11). Early studies, in bacteria and eukaryotes have used similar plasmid-based rDNA *cis*-acting reporter systems to decipher various aspects of the ribosomal subunits life cycle and its regulation (51–63,74,75). This study now provides a unique molecular platform to further unravel archaeal ribosomal biology from an rRNA *cis*-acting element perspective. Importantly, the experimental strategy outlined in this work, may well be applicable to additional genetically tractable model archaea or to improve the read-out of existing bacterial or eukaryotic rDNA *cis*-acting reporter systems.

### A promising scaffold for the engineering of archaeal ribosomal subunits

Ribosome engineering is a gateway to generate synthetic translation machineries with new functional properties enabling, for example, the production of synthetic products or to improve synthesis of proteins of interest (76,77). In the recent years ingenious efforts have been implemented to engineer ribosomal subunits (76,77). This synthetic biology approach has recently culminated by the engineering of functional tethered orthogonal ribosomal subunits based on a similar *cis*-reporter system in bacteria (78–81), thereby opening a new dimension to molecular engineering. Archaea offer several biotechnological advantages as some of them are adapted to extreme conditions (82–85). Accordingly, engineered ribosomal subunits may allow to combine intrinsic properties found across this domain of life and may enable to create synthetic orthogonal ribosomal subunits withstanding extreme conditions required in various biotechnological processes (82–85). We believe that the reporter system described herein has initiated one of the first required steps to generate designer archaeal ribosomal subunits.

### The relevance of circular-pre-rRNA for the proper synthesis of mature rRNAs

The presence of circular pre-rRNA intermediates has been proposed and experimentally confirmed in phylogenetically distant archaea (31,33) (this study – Figure 1). Based on the presence of bulge-helix-bulge motifs within the pre-rRNA processing stems of these organisms, it has also been proposed that the tRNA splicing machinery would be involved in the formation of these archaea-specific pre-rRNA intermediates (31,33,34). However, the biological significance and/or requirement of these circular rRNA intermediates for the efficient synthesis of functional mature rRNA has not been experimentally addressed.

In this study, we have applied a *cis*-acting element perturbation strategy to unravel the functional requirement of rRNA structure/sequence for the synthesis of circular pre-rRNA intermediates and the formation of mature rRNA in *Haloferax volcanii*.

Our investigation suggests that formation of circular-pre-rRNA requires the presence of structurally intact bulge-helix-bulge motifs found in the processing stems generated by inverted sequences flanking both respective mature 16S and 23S rRNA sequences. Moreover, perturbation of circ-pre-rRNA formation is also accompanied by a general decrease of the total rRNA population (note that the mature rRNA is corresponding to >90% of total rRNA) (Figure 7). Together, we suggest that circular-pre-rRNA formation is required for the efficient synthesis of mature functional ribosomal subunits and is a specific feature of archaeal ribosome biogenesis, not so far encountered in bacteria or eukaryotes.

**Figure 8.**
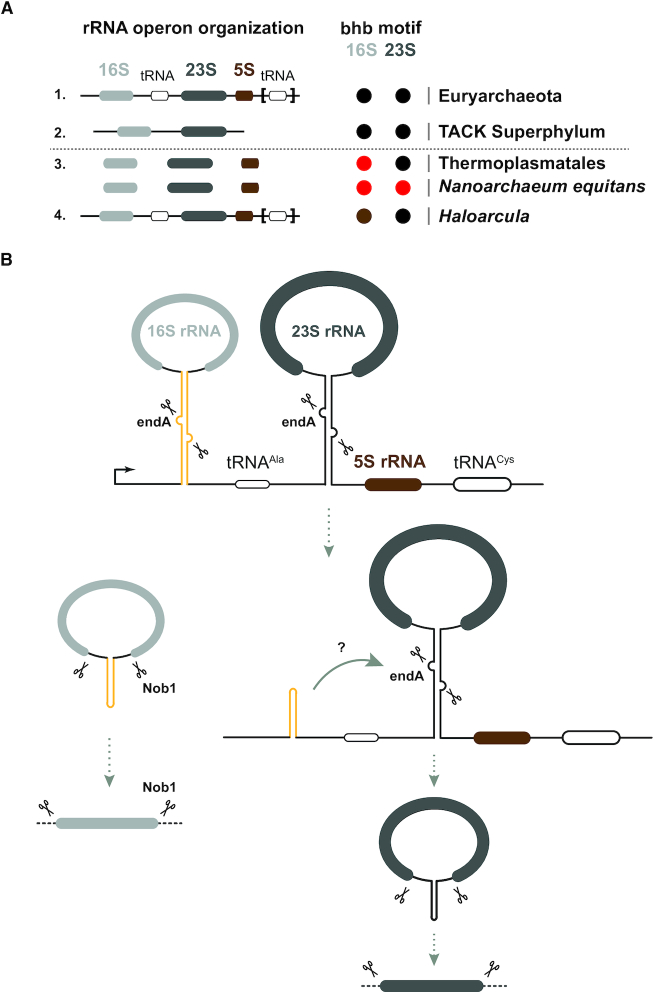
A putative model accounting for functional coordination of early rRNA maturation steps in archaea. (**A**) Ribosomal DNA organization and rRNA bulge-helix-bulge motif conservation across archaea. A selected survey of archaeal rRNA operon organizations suggests 2 predominant classes of linked rRNA organization found in representative organisms of the Euryarchaeota and TACK Superphylum (Thaumarchaeota–Aigarchaeota–Crenarcheota–Korarchaeota) and one minor class of unlinked organization (e.g. *Thermoplasmata* genus order/*Nanoarchaeum equitans*). 16S and 23S rRNAs processing stem secondary structures were predicted using the ViennaRNA Web servers. Presence of predicted bulge-helix-bulge is indicated in black. Presence of heterogeneous rRNA operons with heterogeneous presence of bulge-helix-bulge motif within the processing stem is depicted by a brown circle (*Haloarcula* genus). Absence of predictable bulge-helix-bulge motifs is depicted by a red circle (e.g. *Thermoplasmata* genus/*Nanoarchaeum equitans*). (**B**) Proposed models for early coordination of rRNA maturation in *H. volcanii*. Upon processing of the 16S rRNA processing stem presumably performed by the tRNA splicing machinery, ligation of the processed 16S rRNA flanking region generates circular-pre-16S rRNA intermediates, whereas the remaining processed 5′leader and downstream internal spacer sequences are ligated to the nascent pre-23S rRNA. This ligation event may potentially generate a molecular feature facilitating the subsequent maturation (circularization) of the pre-23S rRNA. Alternatively, rRNA elements upstream of the 23S rRNA processing stem may have an inhibitory effect on the 23S processing stem folding/processing. This inhibitory effect is only alleviated when rRNA elements upstream of the 23S rRNA processing stem are partly or completely removed during early rRNA maturation events (see Discussion for additional details).

How conserved is circular-pre-rRNA formation among archaea? A selected survey of representative organisms of various phyla across the archaeal domain of life, suggests a widespread, however not complete, distribution of the bulge-helix-bulge motif within the pre-rRNA processing stems (Figure 8). For example, *Haloarcula* species possess three divergent rRNA operons (86–88). Interestingly, two of the 16S rRNA processing stems are lacking the bulge-helix-bulge motif (86). Similarly, in *Thermoplasma acidophilum* the 16S rRNA processing stem is lacking the bulge-helix-bulge motif (45,46). In *Nanoarchaeum equitans*, we could not predict any bulge-helix-bulge motif within the putative rRNA processing stems. Whether, these pre-rRNA variants are processed independently of pre-rRNA circularization, utilize non-predicted/cryptic bulge-helix-bulge motifs or a bulge-helix-bulge-independent circularization pathway is intriguing. These observations await a more general and systematic functional analysis aiming to survey biological and functional diversity of ribosome synthesis across archaea.

Remarkably, the utilization of inverted RNA repeats to establish double stranded processing stems is not specific to archaea and is also a general feature found in bacteria. In contrast, to the bulge-helix-bulge motifs, bacterial processing stems commonly contain RNAse III processing sites (89–92). In *E. coli*, absence of RNase III has no significant impact on the formation of mature functional 16S rRNA, whereas 23S rRNA is not fully matured but remains essentially functional, thereby suggesting that RNAse III-dependent cleavage steps are apparently not essential for the efficient cellular accumulation/stability of functional rRNA (91,93–95).

What are the functional advantages of pre-rRNA circularization in archaea?

Among various possibilities and despite the apparent functional differences between archaea and bacteria, it is appealing to consider that processing stem formation enables stabilization and positioning of the future mature 5′-3′-ends in a protected and structurally confined environment, resembling pseudo-circularization. These topological and structural constraints are likely to (i) facilitate assembly and stabilization of early assembling ribosomal proteins, (ii) protect the 5′-3′-ends from exonucleolytic degradation, (iii) stabilize a structural conformation compatible with the following steps of ribosome synthesis and/or inhibiting premature assembly and/or maturation events. The additional covalent circularization step encountered in some archaea may provide an additional safeguard stabilization time-window which is either required in these cellular contexts or has been inherited by a common archaeal ancestor for which pre-rRNA circularization has provided a necessary selective advantage in the early steps of development of the archaeal lineage.

Remarkably, recent structural analyses also suggest that initial steps of eukaryotic pre-rRNA maturation may also resemble, to some extent, pseudo-circularization observed in bacteria/archaea, whereby distant 5′and 3′elements are brought together in a relatively close proximity and stabilized by various molecular mechanisms. Among these, the U3 snoRNP and its associated factors constituent of the SSU processome/90S particles provide an encapsulated environment which also helps to stabilize/hold together distant structural 5′and 3′elements (96–99). In this line, recent studies suggest that the Npa1-complex stabilizes the root helices of LSU domain I and VI, thereby establishing a pseudo-circular structure (100,101). Collectively, these studies suggest that early steps of ribosome synthesis require the formation of a structurally defined protecting environment which is apparently established by pseudo-circularization of the nascent pre-rRNA and which in its general principle seems to follow a rather similar framework across the different domains of life (10).

### Coordination of early rRNA maturation steps in archaea

Our analysis also reveals an intriguing putative coordination of the initial pre-rRNA maturation steps in *H. volcanii*. As summarized in Figure 7, integrity of the 16S rRNA processing stem is both required for the formation of circular and total 16S/23S rRNAs. In contrast, integrity of the 23S rRNA processing stem is only required for the formation of circular and total 23S rRNA and does not significantly affect circular-pre-16S rRNA intermediates or total 16S rRNA levels. Remarkably, this effect also appears to only affect plasmid-derived rRNAs, thereby suggesting a *cis*-acting, plasmid–autonomous, mechanism at the basis of this apparent coordination. Importantly, a previous study from Hüttenhofer and collaborators has suggested that not only the pre-rRNA are ligated but that similar ligation reactions occur at the level of the processed pre-rRNA spacer regions, thereby potentially generating a 5′leader/3′trailer ligation of the processed 16S pre-rRNA flanking regions that still remains attached to the nascent 23S pre-rRNA intermediate (Figure 8) (33). Furthermore, Hüttenhofer and collaborators have also predicted that this ligated region may adopt a C/D box-like snoRNA structure (33). Interestingly, the predicted secondary structure elements within the rRNA spacers regions are also well conserved among archaea (102). However, the *in vivo* functional contribution of this RNA element remains to be determined. Based on these observations, we suggest a model of rRNA maturation in *H. volcanii*, whereby ligation of the resulting processed 16S rRNA 5′leader/3′trailer could potentially serve as a platform recruiting stimulatory activity or facilitating RNA conformational changes required for further maturation of the 23S pre-rRNA. Alternatively, the presence of the ligated spacers and tRNA^Ala^ and/or the not yet processed 16S rRNA/tRNA region upstream of the 23S rRNA processing stem may have an inhibitory effect on the proper folding of the 23S rRNA processing stem and/or on the activity of the intron splicing machinery (Figure 8). Our future work will aim to further disentangle the molecular basis of this apparent functional coordination.

Our results also imply that processing of the tRNA present in between the 16S and 23S rRNA in most Euryarchaeota is rate limited in this cellular context, presumably in order to avoid premature physical separation of the nascent large pre-rRNAs. Remarkably, this possibility is also supported by previous results (33). Moreover, most Crenarchaeota rRNA operons are devoid of an interspersed tRNA between the two large rRNAs, suggesting a putative conservation of this coordination mechanism across a wide range of evolutionary divergent archaea. Interestingly and in stark contrast to the widespread rDNA organizations described above, members of the Thermoplasmatales order and *Nanoarchaeum equitans* possess independent rRNA genes (44,46,47). In addition, no bulge-helix-bulge motifs could be predicted in the 16S rRNA flanking regions of *Thermoplasma acidophilum*, or in both 16S/23S rRNA flanking regions in*N. equitans* (45,46), suggesting a possible functional divergence of the ribosome biogenesis pathway in this particular group of archaea.

Finally, the functional coordination described above may contribute to facilitate stoichiometric production of mature rRNA. However, to our knowledge, functional *cis*-acting or ‘*cis*-acting-like’ coordination in bacteria or eukaryotes has not yet been described. In fact, previous analysis deleting either large part of the rDNA operon or splitting the rDNA operon did not result in strong deleterious effects on the remaining rRNA maturation in bacteria and yeast, respectively (56,62,74). Thereby, these results suggest a lack of coupling of rRNA maturation in *cis*, in contrast to our observations. Moreover, most ribosome biogenesis factors, with a few known exceptions (103–109) are not involved in the maturation of both ribosomal subunits (4–9). Together, these observations suggest that maturation of ribosomal subunits in bacteria and eukaryotes is predominantly occurring independently of each other.

In conclusion, future work will be necessary to further disentangle common and specific molecular principles of ribosome biogenesis across and within the different domains of life.

## Supplementary Material

gkz1156_Supplemental_File

## Notes

Present address: Matthias Weiß, Institute of Epigenetic and Stem Cells - Helmholtz Center Munich - Marchioninistraße 25, 81377 Munich, Germany

Present address: Corinna Reglin, 2bind GmbH, Am Biopark 13, 93053 Regensburg, Germany
