# Supplementary material for: A versatile cis-acting element reporter system to study the function, maturation and stability of ribosomal RNA mutants in archaea
Source: Nucleic Acids Res. 2019 Dec 12;48(4):2073–90. doi: 10.1093/nar/gkz1156 (PMC7038931; doi:10.1093/nar/gkz1156)
Supplement: gkz1156_Supplemental_File [file gkz1156_supplemental_file.pdf]

A versatile *cis*-acting element reporter system to study the function, maturation and stability of ribosomal RNA mutants in archaea

Michael Jüttner, Matthias Weiß, Nina Ostheimer, Corinna Reglin, Michael Kern, Robert Knüppel and Sébastien Ferreira-Cerca

Supplementary Information

Supplementary Table 1-3

Supplementary Figures 1 & 2

Supplementary Table 1. List of Strains used in this study.

Name	Description	Source
Haloferax volcanii - H26	*ΔpyrE2* (derivative from DS70)	(1)
Sulfolobus acidocaldarius (MW001)	*ΔpyrE*	(2)

Supplementary Table 2. List of plasmids used in this study.

Plasmid name	Description	Source
pTA1228	*E.coli – H. volcanii* shuttling vector<br>Amp^R – *pyrE2*	(3)
pRep001	pTA1228 derivative - rDNA locus (HVO_3038-HVO_3042) and flanking region	This study
pRep002	pRep001 derivative - *cis*-acting element reporter<br>$16S^{A633G,C734T}$ $23S^{C2479TA2496C}$	This study
pRep002-$16S^{G468U}$	pRep002 + $16S^{G468U}$	This study
pRep002-16S Δ5' bulge	pRep002 + Δ5' bulge 16S rRNA	This study
pRep002-16S Δ3' bulge	pRep002 + Δ3' bulge 16S rRNA	This study
pRep002-rep 16S ΔΔ bulges	pRep002 + Δ5' bulge Δ3' bulge 16S rRNA	This study
pRep002-23S Δ5' bulge	pRep002 + Δ5' bulge 23S rRNA	This study
pRep002-23S Δ3' bulge	pRep002 + Δ3' bulge 23S rRNA	This study
pRep002-23S ΔΔ bulges	pRep002 + Δ5' bulge 16S rRNA Δ5' bulge 23S rRNA	This study
pRep002-16S Δ5' bulge 23S Δ5' bulge	pRep002 + Δ5' bulge 16S rRNA Δ3' bulge 23S rRNA	This study
pRep002-16S Δ5' bulge 23S Δ3' bulge	pRep002 + Δ5' bulge 16S rRNA Δ3' bulge 23S rRNA	This study
pRep002-16S Δ3' bulge 23S Δ5' bulge	pRep002 + Δ3' bulge 16S rRNA Δ5' bulge 23S rRNA	This study
pRep002-16S helix repeat	pRep002 + helix repeat 16S rRNA	This study
pRep002-16S open helix	pRep002 + open helix 16S rRNA	This study
pRep002-16S stem deletion	pRep002 + processing stem partial deletion 16S rRNA	This study

Details information are available upon request.

Supplementary Table 3. List of oligonucleotides used in this study.

Oligo name	Sequence 5´>3´
oHv039-ci16S-Fw	CGAATCTGGGCTTCGCAAGG
oHv040_ci16S-Rv	GTATGAACTCGTGCAACTAGC
oHv041-ci23S-Fw	CGATAGACTCGGGGTGTACGC
oHv042_ci23S-Rv	CAGCTTGGCACGTCCGTCATC
oHv151-rDNA2860-Fw	GTCTCCTGGAACGGAGCGTG
oHv153-rDNATermNot-Rv	GATCGATGCGGCCGCGGCCAAGACGTAGTCGTCTTGG
oHv154-rDNA4930-Rv	GTTGGGAATGTCACTGTCAGAC
oHv155-rDNA3020-Rv	CAGCGTTCGCTCACGCTACTTG
oHv200-Hv-revS1-001	CCTGCGGTACGCCGCAAGAC
oHv201-Hv-revS3-006	GCAGTACTCCACTCCGAAACG
oHv205_16SrRNA_5' end	ATTCCGGTTGATCCTGCCGG
oHv207-rDNA4099-Fw	CCTTCGCCCCGTCGAATCACC
oHv208-rDNA6008-Rv	ATCGATGCGGCCGCGCGAGAAGTTGAGATCAGCGAGG
oHv209-rDNAPromKpnI ClaI-Fw	GATCCATGGTACCATCGATCTGCCGGCCCACTCATTGCTC
oHv214-23S C2479T Fw	AAGCTACCTTAGGGATAAtAGAGTCGTCACCGGCAAGAGC
oHv215-23S C2479T Rv	GCTCTTGCCGGTGACGACTCTaTTATCCCTAAGGTAGCTT
oHv216-23S A2496C Fw	TAACAGAGTCGTCACCGGCAcGAGCACATATCGACCCGG
oHv217-23S A2496C Rv	CCGGGTCGATATGTGCTCgTGCCGGTGACGACTCTGTTA
oHv220-16S Pacta A633G Fw	GTACGTCCGGGGTAGGAGTGgAATCCCGTAATCCTGGACGG
oHv221-16S Pacta A633G Rv	CCGTCCAGGATTACGGGATTcCACTCCTACCCCGGACGTAC
oHv222-16S Pacta C734T Fw	GTCTCGAACCGGATTAGATAtcCGGGTAGTCCTAGCTGTA

oHv223-16S Pacta C734T Rv	TACAGCTAGGACTACCCGgaTATCTAATCCGGTTCGAGAC
oHv224-16S Pacta C735T Fw	GTCTCGAACCGGATTAGATActCGGGTAGTCCTAGCTGTA
oHv225-16S Pacta C735T Rv	TACAGCTAGGACTACCCGAGTATCTAATCCGGTTCGAGAC
oHv232-16S-RiboTagIFw	CAATGCTACCCTTGAGGTAGCGGCTGGGTACATTAGAAGGACTG
oHv233-16S-RiboTagIRv	CCGCTACCTCAAGGGTAGCATTGCTGGCAATTAGAAGTGCGGGTC
oHv234-23S-RiboTagIFw	CTCACGCGTCGGCGAGAGCCGACGCGAGAGTTCGCTTCCCTGTCAAAC
oHv235-23S-RiboTagIRv	CTCTCGCGTCGGCTCTCGCCGACGCGTGAGTCCGGATTCCCAATCGG
oHv236-rDNA-Pci3462-Rv	GCTTCGACATGTTTCGGTTGGAACC
oHv252-16S-Tag-Tot Rv	TGACTTAACAGGACGCCTCA
oHv255-16S-G468U-Fw	GACCGGTGCCAGCCGCCGCGTTAATACCGGCAGCTCAA GTG
oHv297-Delta16S 5’ Bulge Fw	GTTAGCCCTAGTAGTTCGGTGTCCGAACGGATGTCACGCGAAC
oHv298-Delta16S 5’ Bulge Rv	GTTCGCGTGACATCCGTTCGGACACCGAACTACTAGGGCTAAC
oHv299-Delta16S 3’ Bulge Fw	ATGACACCCGTTCGGACACCGAACTACTAGGGCTAACACGG
oHv300-Delta16S 3´ Bulge Rv	CCGTGTTAGCCCTAGTAGTTCGGTGTCCGAACGGGTGTCAT
oHv301-Delta23S 5´Bulge Fw	GTGTACGTGCAATCCAGGCGTCTGGACCCGTTCTCCGGGTCAC
oHv302-Delta23S 5´Bulge Rv	GTGACCCGGAGAACGGGTCCAGACGCCTGGATTGCACGTACAC
oHv303-Delta23S 3´Bulge Fw	CCGAGAACGGGTCCAGGCGCCTGGATTGCACGGACACATTGG
oHv304-Delta23S 3´Bulge Rv	CCAATGTGTCCGTGCAATCCAGGCGCCTGGACCCGTTCTCGG
oHv305-DY682-16Srep-RV-Fw	GGAAATCCGCCAGCTCAAC
oHv306-DY782-16Srep-RV-Rv	GTACTTCCCAGGCGGCTCG
oHv307-RT-23SrepBss-Rv	CGTTACCTCGTTGCGTACACC
oHv322-DY682-23Srep-Bss-Fw	ACGAGGTTCATTCATGGGAC
oHv323-DY782-23Srep-Bss-Rv	GATAGCAGCCGACCTGTCTC
oHv476_helix repeat 16S 5´_Fw	TAGCCCTAGTAGTTCGGTGGGTGACATCCGAACGGATGT CACGCG
oHv477_helix repeat 16S 5’_Rv	CGCGTGACATCCGTTCGGATGTCACCCACCGAACTACTA GGGCTA
oHv478_helix repeat 16S 3´_Fw	CAATGACACCCGTTCGGACACCCACCTTAGAACTACTAG GGCTAACAC
oHv479_helix repeat 16S 3´_Rv	GTGTTAGCCCTAGTAGTTCTAAGGTGGGTGTCCGAACGG GTGTCATTG
oHv480_open helix 16S 5´_Fw	GATGTTAGCCCTAGTAGTTCAAAAACATCCGAACGGATG TCACGC
oHv481_open helix 16S 5´_Rv	GCGTGACATCCGTTCGGATGTTTTTGAACTACTAGGGCT AACATC
oHv482_processing stem delete 16S 5´_Fw	GCAAGACGGTATCTGATGTTGACCTTTGAACGGTGATTTG
oHv483_processing stem delete 16S 5´_Rv	CAAATCACCGTTCAAAGGTCAACATCAGATACCGTCTTGC
oHv484_processing stem delete 16S 3´_Fw	GTGGCTCACACGCGATCTTCAACACGGGCCCATAGCTCAG
oHv485_processing stem delete 16S 3´_Rv	CTGAGCTATGGGCCCGTGTTGAAGATCGCGTGTGAGCCAC
Saci_009_ci16SrRNA Rv	GAGTGTAAGACTCCCATGGC
Saci_010_ci16SrRNA Fw	GAGAAGTCGTAACAAGGTAG
Saci_013_16SrRNA 5ETS Fw	GATTACGGGTGGTGAAAAGCC
Saci_014_ci23SrRNA Rv	GACCTCGGGGTCAACGGCTGC
Saci_015_ci23SrRNA Fw	CAGGCGTTAGCCGGGGCCTTC
Saci_016_23SrRNA 5´ITS Fw	GTTAGGGCTCAATGAGGCTAG

Supplementary Figure 1

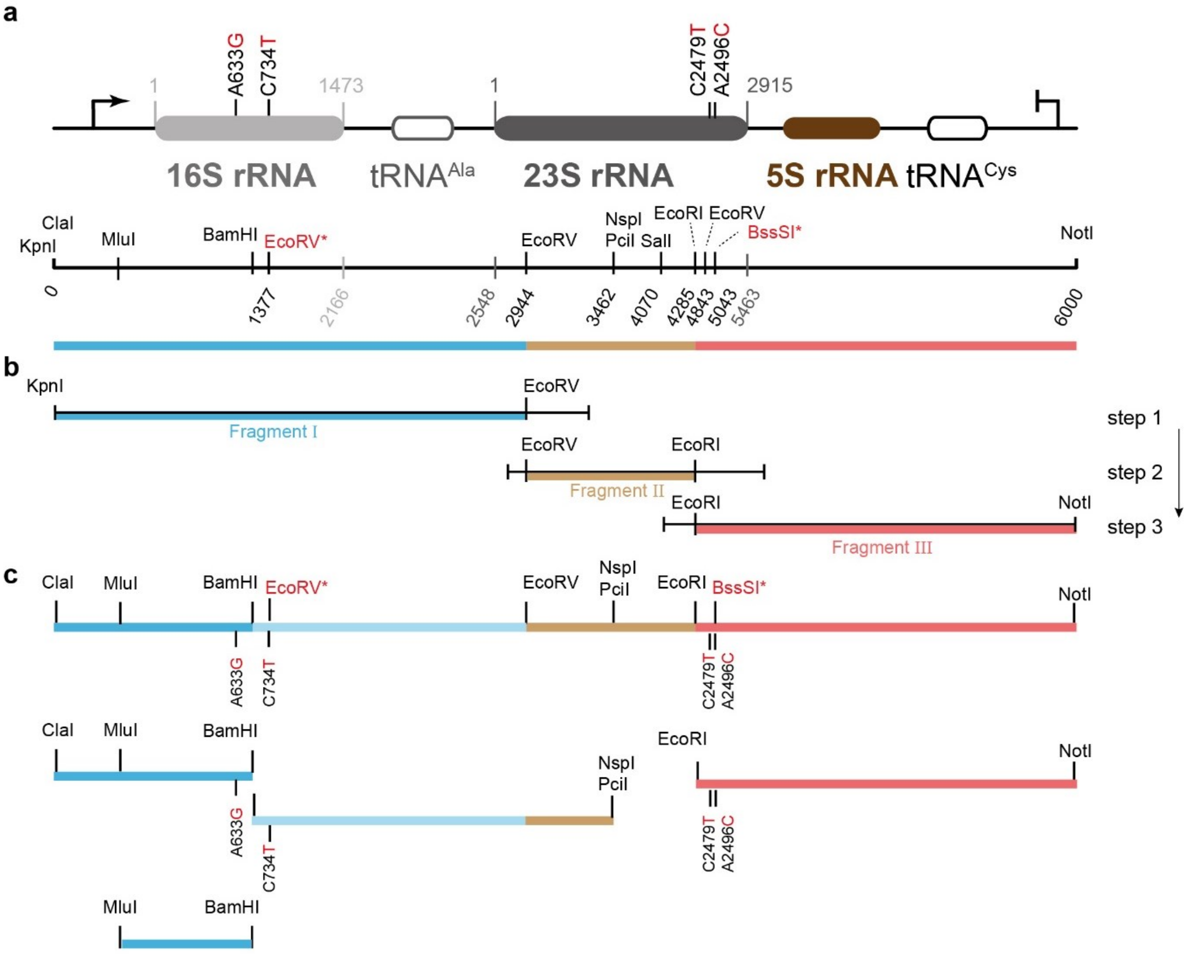

Supplementary Figure 1 | Overview of the cloning strategies of the rDNA operon and its variants.

(a) Schematic representation of the plasmid-based rDNA *cis*-acting element reporter.

Ribosomal DNA operon A (HVO_3038-HVO_3042) and its flanking regions were cloned into pTA1228 generating pRep001. Main restriction sites are indicated with their relative position to the 5´cloned region. *EcoRV*/*BssSI* indicated in red are additional restriction sites generated by the reporter mutations at position 16S^{C734T} (16S rRNA numbering) and 23S^{A2496C} (23S rRNA numbering). The mutated positions 16S^{A633} and 23S^{C2479} providing partial resistance to Pactamycin (16S^{A633G}) and Chloramphenicol 23S^{C2479T}) respectively, are indicated. The colored segments indicate the fragments used for molecular cloning.

(b) Stepwise cloning of the original rDNA operon A using sub-module approach.

Ribosomal DNA operon A (HVO_3038-HVO_3042) and its flanking regions were cloned into pTA1228 generating pRep001, using the indicated 3 steps procedures.

(c) Sub-modules commonly used for insertion of additional mutations within the *cis*-acting element reporter system. Cloning modules and the restriction enzymes typically used for the insertion of bulge-helix-bulge mutations are depicted.

Supplementary Figure 2

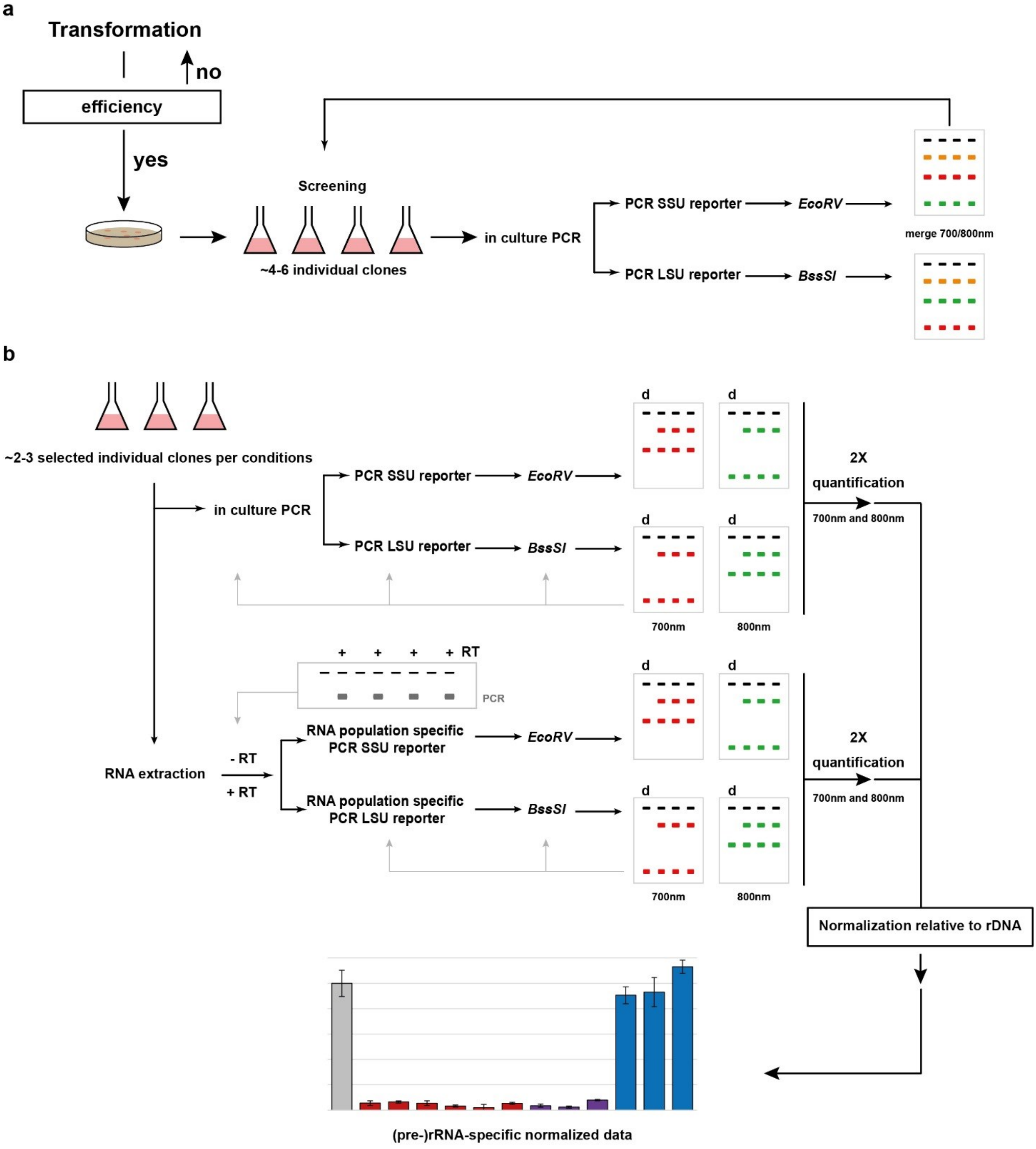

Supplementary Figure 2 | Stepwise workflow overview of the *cis*-acting element reporter system.

(a) Pre-screening of transformants carrying the *cis*-acting element reporter plasmid system. Cells were transformed with the respective plasmid and selected on Hv-Ca$^+$ lacking uracil. Four to six individual clones were first used to confirm the homogenous presence and integrity of the reporter system at the DNA level.

(b) Standard data collection workflow.

From the verified cloned in **(a)**, 2-3 independent clones were cultivated and subjected to the *cis*-reporter element standard workflow. In culture PCR was performed using SSU/LSU reporter fluorescent PCR primers and digested with *EcoRV* or *BssSI,* respectively. Total RNA was extracted, and DNase treated, before cDNA synthesis using rRNA intermediates-specific primers. PCR was performed using SSU/LSU reporter fluorescent PCR primers. Samples were further digested only if no PCR products was observed in the no reverse transcriptase control. All digested samples were separated by PAGE and fluorescent signals (700nm and 800nm) were acquired using a Li-COR odyssey imaging platform. All results were quantified twice and at two different wavelength. Relative rRNA expression was normalized to the relative rDNA amounts. The wildtype results used as reference and arbitrarily set to one.

Note that digest controls, labelled “d”, were incorporated to each digestion to verify the completeness of the restriction digest reaction (see **Materials and Methods**)

Supplementary References

1. Allers,T., Ngo,H.-P., Mevarech,M. and Lloyd,R.G. (2004) Development of Additional Selectable Markers for the Halophilic Archaeon Haloferax volcanii Based on the leuB and trpA Genes. *Applied and Environmental Microbiology*, **70**, 943–953.

2. Wagner,M., van Wolferen,M., Wagner,A., Lassak,K., Meyer,B.H., Reimann,J. and Albers,S.-V. (2012) Versatile Genetic Tool Box for the Crenarchaeote Sulfolobus acidocaldarius. *Frontiers in Microbiology*, **3**, 214.

3. Brendel,J., Stoll,B., Lange,S.J., Sharma,K., Lenz,C., Stachler,A.-E., Maier,L.-K., Richter,H., Nickel,L., Schmitz,R.A., *et al.* (2014) A Complex of Cas Proteins 5, 6, and 7 Is Required for the Biogenesis and Stability of Clustered Regularly Interspaced Short Palindromic Repeats (CRISPR)-derived RNAs (crRNAs) in Haloferax volcanii. *Journal of Biological Chemistry*, **289**, 7164–7177.
